# Olive Leaves as Biotemplates for Enhanced Solar-Light Harvesting by a Titania-Based Solid

**DOI:** 10.3390/nano10061057

**Published:** 2020-05-30

**Authors:** Jesús Hidalgo-Carrillo, Juan Martín-Gómez, M. Carmen Herrera-Beurnio, Rafael C. Estévez, Francisco J. Urbano, Alberto Marinas

**Affiliations:** Departamento de Química Orgánica, Instituto Universitario de Investigación en Química Fina y Nanoquímica (IUNAN), Universidad de Córdoba, Campus de Rabanales, Edificio Marie Curie, E-14071 Córdoba, Spain; juanmartingomez@outlook.es (J.M.-G.); b52hebem@uco.es (M.C.H.-B.); q72estor@uco.es (R.C.E.); alberto.marinas@uco.es (A.M.)

**Keywords:** olive leaf, biotemplating, titania-based materials, textural and structural characterization, solar-light harvesting, photocatalysis, hydrogen production

## Abstract

Olive leaves (by-product from olive oil production in olive mills) were used as biotemplates to synthesize a titania-based artificial olive leaf (AOL). Scanning electron microscopy (SEM) images of AOL showed the successful replication of trichomes and internal structure channels present in olive leaves. The BET surface area of AOL was 52 m^2^·g^−1^. X-ray diffraction (XRD) and Raman spectra revealed that the resulting solid was in the predominantly-anatase crystalline form (7.5 nm average particle size). Moreover, the synthesis led to a red-shift in light absorption as compared to reference anatase (gap energies of 2.98 and 3.2 eV, respectively). The presence of surface defects (as evidenced by X-ray photoelectron spectroscopy, XPS, and electron paramagnetic resonance spectroscopy, EPR) and doping elements (e.g., 1% nitrogen, observed by elemental analysis and XPS) could account for that. AOL was preliminarily tested as a catalyst for hydrogen production through glycerol photoreforming and exhibited an activity 64% higher than reference material Evonik P25 under solar irradiation and 144% greater under ultraviolet radiation (UV).

## 1. Introduction

Bio-inspired materials are a promising research area for the development of advanced systems with higher environmental compatibility, recyclability and energetic efficiency [1]. Some of the advantageous properties of natural materials include sophistication, miniaturization, hierarchical organization, resistance and adaptability, which are the result of billions of years of evolution [2]. The morphologies of biological structures ranging from nanometers to the millimeter scale can inspire the design of artificial materials to be used for energy capture, storage and conversion [3,4,5].

The use of natural materials as biotemplates to obtain heterogeneous catalysts is effective in overcoming the difficulty of controlling the morphology of the final solid [6,7,8]. The main advantage of this approach is that the final structure can be easily designed by the selection of a template with a specific morphology, while conventional synthesis processes, such as the sol-gel method, are greatly affected by the synthetic conditions, such as the pH of the solution, the drying process, subsequent heat treatments, etc.

Hydrogen is considered to be an efficient source of non-polluting energy, with an acceptable cost in the medium and long term. Currently, hydrogen is mainly produced by steam reforming and water electrolysis [9]. The former approach has the disadvantage of using non-renewable fossil sources and, therefore, the co-generated CO_2_ directly impacts the environment through the greenhouse effect. A second drawback of steam reforming is its high operating temperature. As for hydrogen production through water electrolysis, its main associated problem is the high consumption of electrical energy [9]. Alternatively, solar thermal energy can be used but then large and expensive facilities are required.

In addition to the above-mentioned technologies for hydrogen production, some innovative techniques are being developed that could be complementary to those already existing in the medium-term future. Among them, photocatalytic reforming of oxygenated organic compounds is one of the most promising. It consists in the treatment of such compounds with light radiation in the presence of water, at room temperature and anaerobic conditions, to generate gaseous hydrogen and carbon dioxide [10]. The process is particularly interesting from the environmental point of view if biomass residues or by-products (bio-glycerol or glucose, among others) are used as oxygenates since, in this case, the CO_2_ generated is consumed by the biomass itself during its growth, so the carbon cycle is closed. One of the key points to the success of this technology is the development of some suitable catalysts (i.e., semiconductors) able to maximize the light harvesting and therefore the hydrogen production [11]. Titania is the most widely used semiconductor due to its excellent stability during the photochemical process. However, it has several drawbacks, such as an Eg > 3.0 eV, which limits its capacity to absorb visible radiation and a high electron hole recombination rate. In recent years, some intense research has been developed aimed at improving the photocatalyst in this type of process. There are two main ways to retard the electron-hole recombination in TiO_2_: (i) the generation of irregularities or surface defects, or (ii) the modification of TiO_2_ with a metal, creating a Shottky barrier in the junction zone between the two materials that would limit the recombination process, by flowing electrons generated from the valence band of TiO_2_ to the metal [12]. Noble metals such as Pt, Pd or Au [10,13] have been found to be particularly effective though there is a need to use some more cost-effective transition metals such as Fe, Cu or Ni [14,15,16]. This barrier can also be created by modifying TiO_2_ with carbon nanostructures, such as nanotubes or graphene [17]. Another strategy is to couple TiO_2_ with a semiconductor having a smaller band gap to form a heterostructure. This way, heterojunction promote the separation of photoexcited electron hole pairs through several carrier-transfer pathways by keeping reduction and oxidation reactions at two different reaction sites. Moreover, the coupled semiconductors can extend the light response range to the visible [18].

Recent studies on the use of bio-templates for the synthesis of titania-based catalysts have led to solids operating in the visible range [19,20]. Therefore, for instance, Mohamed et al. [21] developed mesoporous nanotubes of TiO_2_ replicating the cellulose structure, thus obtaining a catalyst with high catalytic efficiency in removal of organic pollutants from wastewater when illuminated with visible light. In a different approach, Hashemizadeh et al. [22] described a procedure whereby Camellia leaves were modified through incorporation of TiO_2_ and subsequently of RuO_2_. The use of the plant’s light collection system (mesoporous structures imitating the pores in green leaves) allowed the resulting solid to exhibit better results in CO_2_ reduction or ethanol reforming than Evonik P25 itself (a reference titania photocatalyst), the artificial TiO_2_-based leaves even operating under visible light. These studies are a source of inspiration for some other approaches aimed at valorizing different regional biomass by-products which could contribute to the economic development of involved regions.

The European Union (EU) is the leading world producer of olive oil (roughly 80% of the total output with 37% of the global figure coming from Andalusia, where ca. 250,000 families live on olive cultivation). One of the by-products obtained in the oil mills is the olive leaf. During the previous process of cleaning the olive, a considerable amount of olive leaves is separated representing ca. 8% by weight of the milled olive. Those leaves can be used for livestock feed (although its use is limited by the bad taste that confers the olive juice), as a fuel in power generation plants [23] and as biomass source for obtaining nutraceuticals [24] or potential drugs [25].

In the present piece of research, the structure of natural olive leaves has been replicated for the synthesis of new TiO_2_ structures capable of capturing visible light. Furthermore, as a proof of concept, some preliminary results on hydrogen production through glycerol (bioproduct from biodiesel obtaining) photoreforming are presented.

By this way, a titania-based biomimetic material (artificial olive leaf, AOL) has been synthesized with enhanced optical properties (i.e., light absorption extended to visible) and with a larger amount of interstitial defects than reference Evonik P25. Thus, the electron-hole recombination rate would be reduced and photo-catalytic performance enhanced, as proved in the aqueous glycerol photoreforming leading to hydrogen generation.

## 2. Materials and Methods

### 2.1. Synthesis of Artificial Olive Leave (AOL)

**Figure 1:**
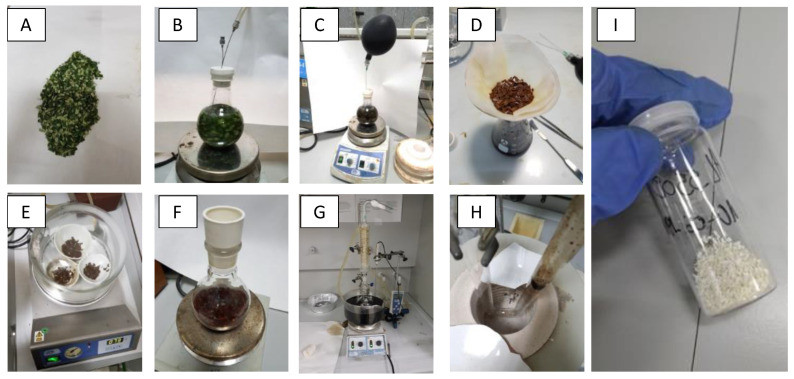
Pictures taken at different steps during the synthesis of AOL. (**A**) Fresh olive leaves cut and washed. (**B**) Acid hydrolysis under N_2_ atmosphere. (**C**) Addition of TiCl_3_ under N_2_ atmosphere. (**D**) Filtering and washing. (**E**) Drying at 120 °C. (**F**) Suspension of leaves in isopropanol and titanium isopropoxide. (**G**) Reflux at 80 °C, 6 h. (**H**) Calcination at 550 °C. (**I**) Resulting AOL.

The replication of architectures of olive leaf was done according to the method described by Li et al. [19] slightly modified. Figure 1 includes several pictures taken at the different steps of the synthetic method. In short, 10 g of fresh olive leaves were cut and washed with Milli-Q water and subsequently treated, under inert (N_2_) atmosphere, with a 0.5% (v/v) solution of HCl until the leaf coloration changed from green to brown. Then, 30 mL of 15% TiCl_3_ water solution (Sigma-Aldrich, Darmstadt, Germany) was added to carry out the ionic exchange and left under vigorous stirring overnight. The leaves were then filtered and washed with Milli-Q water 3 times and left to dry in a vacuum desiccator. The dry leaves were suspended in 100 mL of isopropanol (Merck, Hunterdon County, NJ, USA) for 12 h under stirring, to remove traces of water. They were subsequently filtered and suspended again in 100 mL of isopropanol, 9 mL of titanium isopropoxide (Sigma-Aldrich, Darmstadt, Germany) were added and the suspension was left stirring overnight. Finally, the suspension was refluxed at 80 °C for 6 h, the solid was filtered, dried at 120 °C and calcined at 550 °C for 6 h thus obtaining the AOL (standing for Artificial Olive Leaves). In addition to AOL, two commercial titania materials were used for comparative purposes: Anatase (Sigma-Aldrich, Darmstadt, Germany) and Aeroxide^®^ P25 (Evonik, Essen, Germany).

### 2.2. Characterization of Artificial Olive Leave (AOL) 

X-ray fluorescence analyses was carried out on a Rigaku tube-above wave-length dispersive X-ray fluorescence ZSX Primus IV spectrometer (Rigaku, Austin, TX, USA), equipped with an X-ray tube with 4 kW rhodium anode, a proportional gas flow detector for light elements and a scintillation counter for heavy elements.

The elemental analysis for the AOL solid was carried out at the Central Service for Research Support (SCAI) of the University of Córdoba, using a Eurovector EA-3010 elemental analyzer (Eurovector, Pavia, Italy). 

Scanning electron microscopy (SEM) and energy-dispersive spectroscopy (EDS) analysis were obtained at the Central Service for Research Support (SCAI) of the University of Córdoba with a JEOL JSM 7800F microscope interfaced to an Oxford Instruments X-max 150 semi-quantitative elemental microanalyzer (Jeol, Tokyo, Japan).

Nitrogen adsorption-desorption isotherm, at the liquid nitrogen temperature, were performed on an Autosorb-iQ-MP/MP-XR device (Anton Paar, Graz, Austria), using the Brunauer–Emmet–Teller (BET) method for the calculation of the surface area, the BJH method for the calculation of the area and the volume of mesopores and macropores and the *V*-*t* method for the calculation of the area and pore volume of micropores. Before measurements, all samples were degassed at 120 °C and 0.1 Pa.

XRD analysis were performed on a Bruker D8 Discover (Bruker Española S.A., Madrid, Spain) with a monochromatic CuKα1 radiation (*λ* = 1.54 Å) over an angular range of 10–80° at a scan speed of 1.45° 2*Ɵ*·min^−1^.

Raman spectroscopy was carried out at the Central Service for Research Support (SCAI) of the University of Córdoba on a confocal NRS-5500 Raman spectrometer (Jasco Inc., Tokyo, Japan) with 532 nm laser excitation, L1800 grating and an EMCCD detector. Spectra were taken through 20× objective lens, accumulating 10 scans (10 s exposure) with a laser power at sample point of 0.7 mW.

Diffuse reflectance UV-Vis spectra were performed on a Cary 1E (Agilent, Santa Clara, CA, USA) instrument, using polytetraethylene as reference material. Band gap (Eg) values were obtained from the plot of the modified Kubelka–Munk function [F(R)·E]^1/2^ versus the energy of the absorbed light E. 

X-ray photoelectron spectroscopy (XPS) data was recorded at the Central Service for Research Support (SCAI) of the University of Córdoba on pellets after outgassing the samples to a pressure below 2 × 10^−8^ Torr at 150 °C. A Leibold–Heraeus LHS10 spectrometer (SPECS, Berlin, Germany) was operated with the AlKα (*hν*  =  1486.6 eV) X-ray source at 120 W and 30 mA using C (1 s) as energy reference (284.6 eV).

Electron paramagnetic resonance (EPR) spectra were recorded on a Bruker EMX micro (Bruker, Española S.A, Madrid, Spain) applying and X-band (9.43 GHz, 1.5 mW) microwave with sweeping magnetic field at −173 °C.

### 2.3. Photo-Catalytic Experiments

The liquid-phase photocatalytic reactions were performed in two different devices using suspensions of 1 g·L^−1^ catalyst in 10% (v/v) glycerol in water solutions under inert atmosphere.

**Figure 2:**
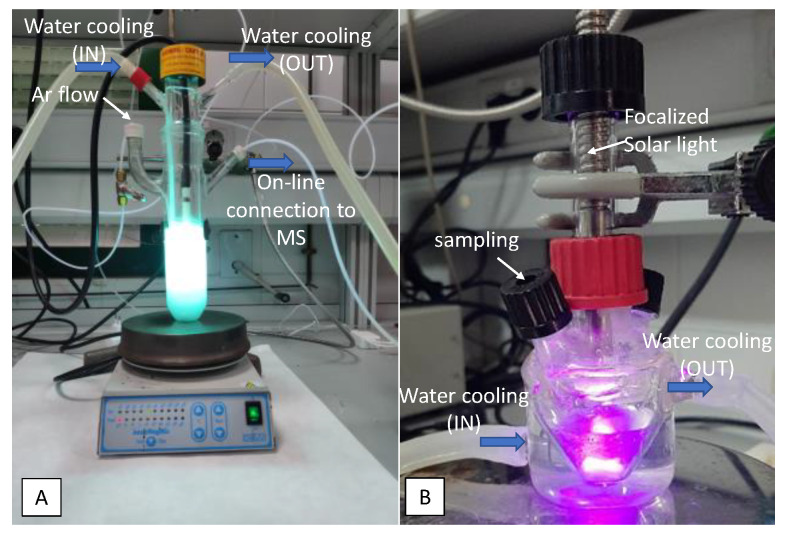
Picture of the photocatalytic reactors used in the hydrogen production from glycerol photo-reforming. (**A**) UV-light. (**B**) Solar light.

For experiments under UV light, the device consisted in a Pyrex cylindrical doubled-walled immersion well reactor (23 cm × 5 cm internal diameter, with a total volume of 190 mL) equipped with a gas circulation system (Ar, 5 mL·min^−1^), and a medium pressure 125 W-Hg lamp (Photochemical Reactors Ltd., Reading, UK), used as the excitation source (Figure 2A). During the process, Ar was bubbled through the suspension (20 mL·min^−1^). Outlet gas composition was on-line analyzed by mass spectrometry.

For solar-light experiments, reactions were performed under nitrogen atmosphere in a 30 mL double mouthed heart-shaped reactor with light (from a Newport solar simulator furnished with a 150 W xenon lamp) focalized on the sample compartment through an optic fiber (Figure 2B). Analyses were performed by sampling with a pressure-lock precision analytical syringe (Valco VICI Precision Syringes, 1 mL, leak-tight to 250 psi) from the head space of the photoreactor at selected times. Samples were analyzed by gas chromatography with a thermal conductivity detector (GC-TCD) on an Agilent Technologies 7890A gas chromatograph (Agilent, Santa Clara, CA, USA) furnished with a Supelco Carboxen™ 1010 Plot column (Sigma-Aldrich, Darmstadt, Germany). 

Lamps power at the sample compartments as measured at <800 nm with an Ophir Starlite equipment were 116 mW·cm^−2^ and 106 mW·cm^−2^ for UV lamp and solar simulator, respectively.

## 3. Results and Discussion.

### 3.1. Olive Oil Leaf Structure

**Figure 3:**
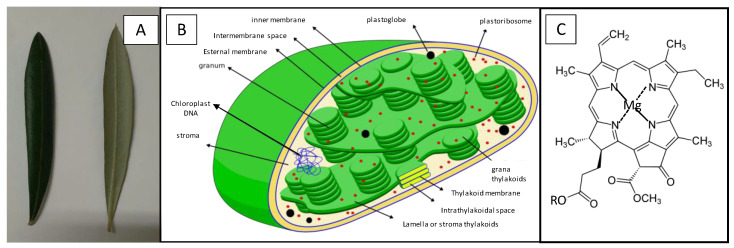
(**A**) Picture of olive leaves. (**B**) Structure of chloroplast, responsible for photosynthesis (Source: Miguelsierra/CC BY-SA (https://creativecommons.org/licenses/by-sa/4.0)). (**C**) Chlorophyll A structure where R denotes the phytol chain.

**Figure 4:**
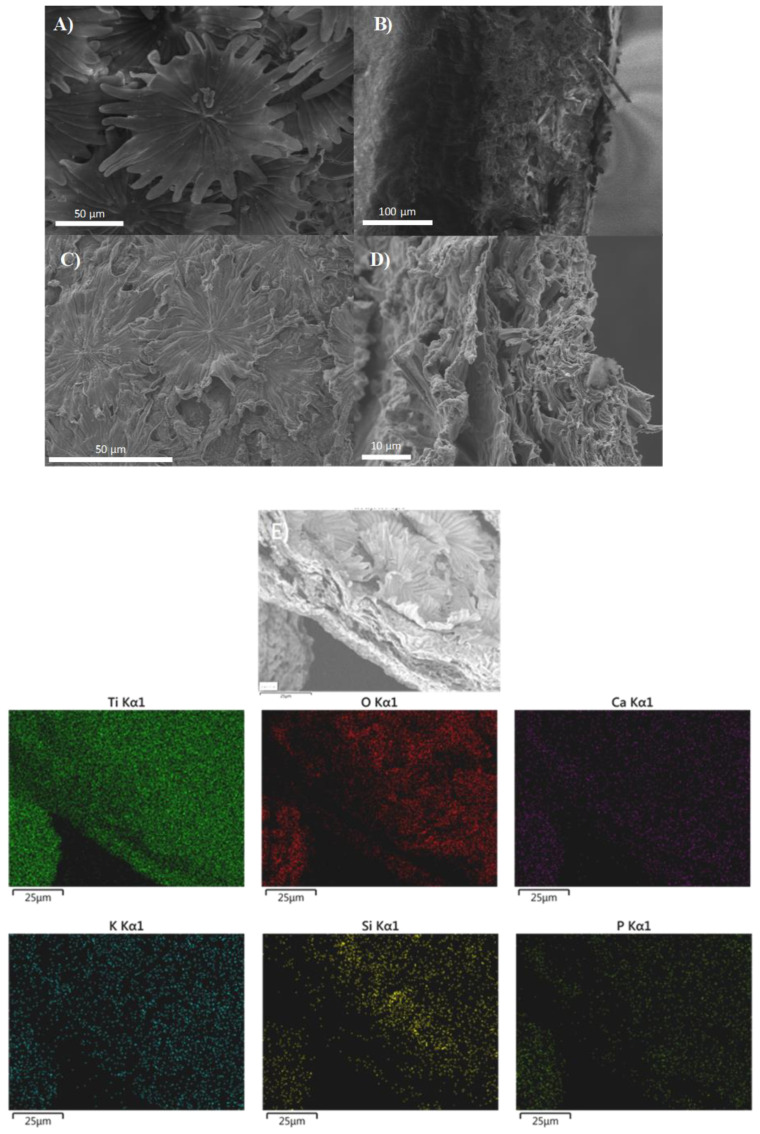
SEM images of a fresh olive leaf (**A**,**B**) and the artificial olive leaf (**C**,**D**). (**E**) Elemental mapping (SEM-EDX) of AOL sample indicating the distribution of Ti, O, Ca, K, Si and P.

Olive leaves (Olea europaea) are evergreen leaves with a 3–9 cm long and 1–2 cm wide limb, a very marked central nerve and a very short petiole (Figure 3A). The epidermis has a cuticle where the stomata are observed, much more abundant on the underside. The stoma is protected by scaly umbrella-shaped hair (trichomes, Figure 4A) whose function is to avoid the loss of water by evaporation. Underneath the epidermis the palisade parenchyma can be found consisting of three compact layers of cells superimposed on the beam and one layer on the underside. Inside the parenchyma cells of the olive leaves there are chloroplasts (Figure 3B), which are organelles in which photosynthesis is performed. Inside the chloroplasts there are thylakoids, flattened sacs delimited by a membrane and stacked forming structures called grana (granum), with a diameter between 0.3–0.6 µm. These stacks are connected laterally to each other by membranes, forming a continuous compartment within the chloroplast. The chloroplast space surrounding the thylakoids is called stroma.

The main mission of chloroplasts is to develop photosynthesis, that is, the conversion of electromagnetic sunlight energy into chemical energy through chlorophyll molecules, adenosine triphosphate (ATP) synthase and ribulose biphosphate carboxylase/oxygenase. The thylakoid membrane contains the molecules responsible for performing the light phase of photosynthesis (photochemical phase), while in the stroma surrounding the thylakoids the dark (non-luminous) phase of photosynthesis takes place.

Chlorophyll molecules (Figure 3C) are formed by a hydrophobic phytol chain and a magnesium containing porphyrin ring, which acts as a light collector during photosynthesis [26]. 

All in all, nature has developed complex systems for light harvesting over millions of years of evolution which are a source of inspiration when planning the design of efficient systems to capture light.

### 3.2. Synthesis of Artificial Olive Leaves (AOL)

Artificial olive leaf (AOL) was synthesized by mimicking the structure of the olive leaf, following the procedure described by Li et al. [19] and subsequently modified by Hashemizadeh et al. [26]. The process basically consists of three phases, (i) an acid treatment by which several metal cations such as Ca, P, S, K could be (partially) eliminated and, especially, Mg from the porphyrins, can be replaced by H^+^, forming yellow-brown pheophytins; (ii) a second step in which these protons are exchanged for Ti^+3^ through a treatment with TiCl_3_ and (iii) a third step in which these Ti^+3^ ions act as seeds for the subsequent formation of a TiO_2_ structure that replicates that of the olive leaf. To achieve the optimal acid treatment, several tests were carried out submitting the olive leaves to HCl solutions of different concentrations (0.5%, 1% and 5%). The use of 1% and 5% HCl resulted in total degradation of the olive leaf while with 0.5% HCl the desired partial degradation of the leaf was achieved, obtaining the brown color indicative of magnesium-proton exchange in porphyrins.

### 3.3. Chemical Analysis of AOL

During biotemplating treatment, ca. 90% of the leaf weight was lost. Table 1 shows AOL elemental composition as determined by X-ray fluorescence (XRF). For comparative purposes, composition of fresh olive leaves reported by Alcázar Román et al. [27] have also been included. As expected, titanium is the major element in AOL, accounting for 53.2% of weight (which expressed as TiO_2_% would represent ca. 89% of the catalyst weight). Other elements detected in AOL by XRF were calcium (1.83%), phosphorus (1.09%) and potassium (0.95%). Those elements were already present in olive leaf. Complementary results using a CHN elemental analyzer, showed that AOL had a carbon content of 1.58% and a nitrogen content of 1.03%. Thus, the C/N weight ratio in AOL is much lower than that described in olive leaves [28] (1.5 and 33, respectively). This suggests that acid treatment and subsequent calcination of olive leaves led to the loss of most of carbon content whereas nitrogen present in the samples remained to a larger extent.

### 3.4. Morphology of the Synthesized AOL

To evaluate the degree of emulation of the micro and/or nanostructure of the olive leaf in the synthesized material, AOL was analyzed by scanning electron microscopy (SEM). Figure 4 shows SEM images of a fresh olive leaf (A and B) and AOL (C and D). Figure 4A is the underside of the olive leaf with the typical trichomes which were successfully replicated in AOL (Figure 4C).

Moreover, Figure 4B,D correspond to a cross-section of the olive leaf and the AOL catalyst, respectively. In these images, the internal structure corresponding to the parenchyma can be independently observed with the channels also replicated in the process. Dehydration and extraction of organic matter resulted in a contraction of the original microstructure of the olive leaf (compare Figure 4A vs. Figure 4C and Figure 4B vs. Figure 4D).

EDX analysis of AOL confirmed that it is mainly formed by titanium. Furthermore, some other minor elements were detected, such as Ca, P, K and Si, in line with that found by XRF. Figure 4E includes a SEM-EDX elemental mapping of different elements present in AOL. As can be seen, titanium is homogeneously distributed in the sample.

### 3.5. Textural Characterization of AOL

**Figure 5:**
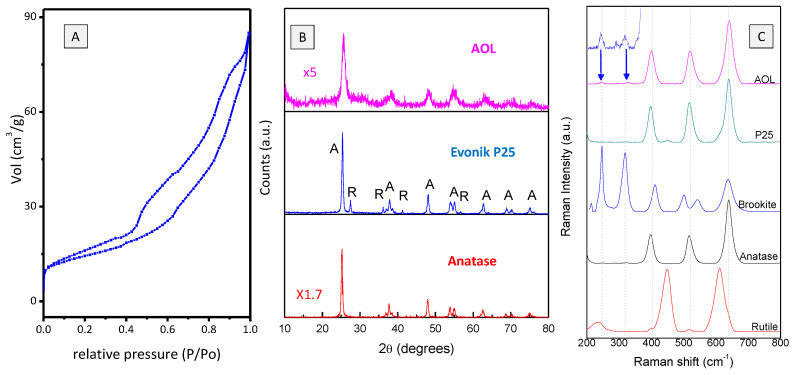
Characterization of AOL. (**A**) Nitrogen adsorption-desorption isotherm. (**B**) XRD pattern. Those patterns of Evonik P25 and Anatase have also been included for comparison. Anatase (A) and Rutile (R) peaks are marked for Evonik P25. (**C**) Raman spectra. For comparative purposes, spectra of brookite and rutile reference materials have also been included. Spectrum of brookite was obtained from RRUFF database (https://rruff.info/brookite/) whereas that of rutile corresponds to a reference material from Sigma-Aldrich (Sigma-Aldrich, Darmstadt, Germany).

N_2_ adsorption-desorption isotherm of AOL (Figure 5A) is type IV, associated to mesoporous materials. Surface area is 52 m^2^·g^−1^, a similar value to that of reference material Evonik P25 (51 m^2^·g^−1^) [29], whereas for anatase (Sigma-Aldrich, Darmstadt, Germany,) the surface area is ca. 45 m^2^·g^−1^ [30]. BJH method showed that macroporous and mesoporous in AOL accounts for 46.36 m^2^·g^−1^ (pore volume of 0.128 cm^3^·g^−1^) whereas microporous have a surface area of 5.64 m^2^·g^−1^ and a volume of 0.002 cm^3^·g^−1^.

### 3.6. Structural Characterization of AOL

Figure 5B shows the X-ray diffractograms of AOL, Evonik P25 and anatase. Evonik P25 (80% anatase/20% rutile) exhibits some characteristic peaks at 2*θ* values of 25.37°, 37.86° and 48.12° corresponding to (101), (004) and (200) lattice planes in anatase and at 27.52° and 36.18° associated to (110) and (101) plane in rutile [31,32]. In the case of AOL only anatase reflections are observed at 2θ values of 25.64°, 38.21°, 48.14°, 54.75°, 63.30°, 69.54° and 75.48° assigned, respectively, to (101), (004), (211), (204), (116) (220) and (215) reflections [32].

It has been reported that pure TiO_2_ calcined at 500 °C exhibits the crystalline anatase phase [33] while the rutile phase begins to develop upon calcination at temperatures from 550 °C. Nevertheless, the presence of additives retards the crystallization of the anatase phase and its transformation into rutile [34]. In our case, the presence of trace elements from the olive leaf (evidenced by XRF) could act in this direction and, therefore, for the AOL material calcined at 550 °C, wide peaks corresponding to the anatase phase are observed, probably associated with a delayed crystallization of the material.

Average crystallite sizes of TiO_2_ were determined applying the Scherrer formula using the (101) and (110) diffraction peaks for anatase and rutile, respectively. Therefore, anatase crystallite sizes of 7.5, 20 and 21 nm were found for AOL, anatase and P25, respectively. Rutile crystallite size in P25 is 39 nm. Crystallite sizes for P25 are consistent with those reported in the literature [30].

Identification of crystalline phases of TiO_2_ in samples was also performed by Raman spectroscopy, which is more sensitive than XRD [34] (Figure 5C). AOL exhibited three bands at 399.8, 520.8 and 640.6 cm^−1^ attributed to active modes of anatase with the symmetries B1g, A1g and Eg, respectively [32,35], while no bands associated to rutile phase were detected. Moreover, unlike XRD, Raman allowed us to identify some very small brookite signals at 245.9 and 328.1 cm^−1^ (A1g and B1g, respectively) [36]. In the case of Evonik P25, Raman spectroscopy confirmed the additional presence of rutile, with a characteristic signal at 446.8 cm^−1^ (Eg symmetry) [37].

Morphological and structural characterization indicates that the micro and nano structure of the olive oil has been successfully replicated and that the obtained solid has a granular morphology consisting of small crystallites of anatase (7.5 nm), and brookite to a lesser extent, with channels interconnecting internal structures, leading to a high surface area solid.

**Figure 6:**
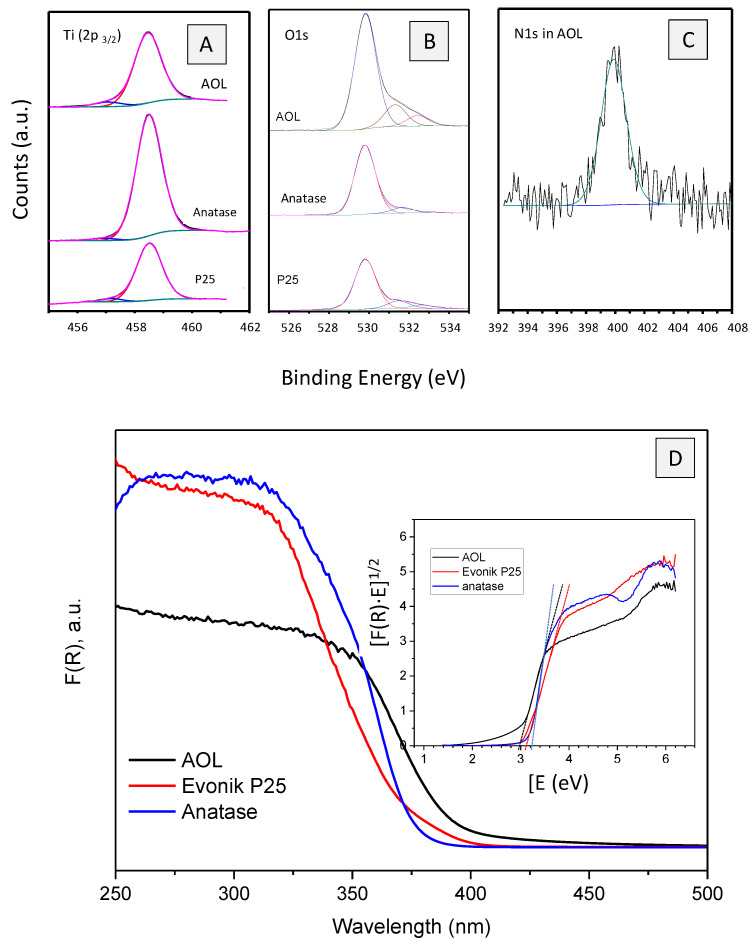
Characterization of AOL. X-ray photoelectron (XPS) profiles of Ti 2p_3/2_ (**A**), O 1s (**B**) and N 1s (**C**). Titanium and oxygen profiles of anatase and Evonik P25 have also been included for comparison. (**D**) UV-Vis spectra of AOL, anatase and Evonik P25. The inset represents the plot of transformed Kubelka–Munk function vs. the energy of the absorbed light for determination of the band-gap energy.

The solids were also analyzed by XPS. Ti2p3/2 signal (Figure 6A) can be deconvolved in two peaks at ca. 458.5 eV and 457.0 eV which are associated to Ti^4+^ and Ti^3+^, respectively [38,39,40,41]. The latter signal represents 1.9%, 4.3% and 6.5% of titanium signal in anatase, P25 and AOL, respectively. O1s spectra of the samples (Figure 6B) exhibit three signals centered at 529.8, 531.3 and 532.6 eV which are assigned in the literature to lattice oxygen atoms, surface hydroxyl groups and chemisorbed O_2_, respectively. The peak at ca. 532.6 eV is associated to the existence of surface defects (such as oxygen vacancies) for O_2_ absorption [42]. It represents 4.3%, 5.4% and 7.2% of oxygen signal in anatase, P25 and AOL, respectively. XPS confirmed the presence of nitrogen in AOL (0.96 wt %). Furthermore, N1s signal is centered at 400 eV (Figure 6C) which is associated to interstitial N-doping [43].

UV-Vis spectra of the solids are presented in Figure 6D. As can be seen, absorption of AOL is shifted to the visible range as compared to P25 and anatase. Therefore, band gaps of 2.98, 3.12 and 3.22 eV, respectively, were found (see inset in Figure 6D). The absorption of visible light by AOL could be the result of a synergistic effect of defects (such as oxygen vacancies) and nitrogen species [44,45] which were found to a larger extent in AOL than in anatase and P25. Oxygen vacancies can give rise to local states below the conduction band. Therefore, the excitation of electrons from the valence band to the oxygen vacancy level results in typical excitations in the visible range. Furthermore, electrons in the oxygen vacancies can interact with adjacent Ti^4+^ and lead to Ti^3+^ species which form a shallow donor level below the conduction band thus also contributing to the visible light response. XPS confirmed that Ti^3+^ species were particularly abundant in AOL. Moreover, oxygen vacancies are stabilized by doping nitrogen. N doping at levels up to 1% can also lead to visible light response because of the N-2p narrow band above the O-2p valence band [46]. The contribution of some other dopant elements which were already present in the olive leaves (e.g., P, C) [47,48] to the redshift of light absorption in AOL cannot be ruled out.

**Figure 7:**
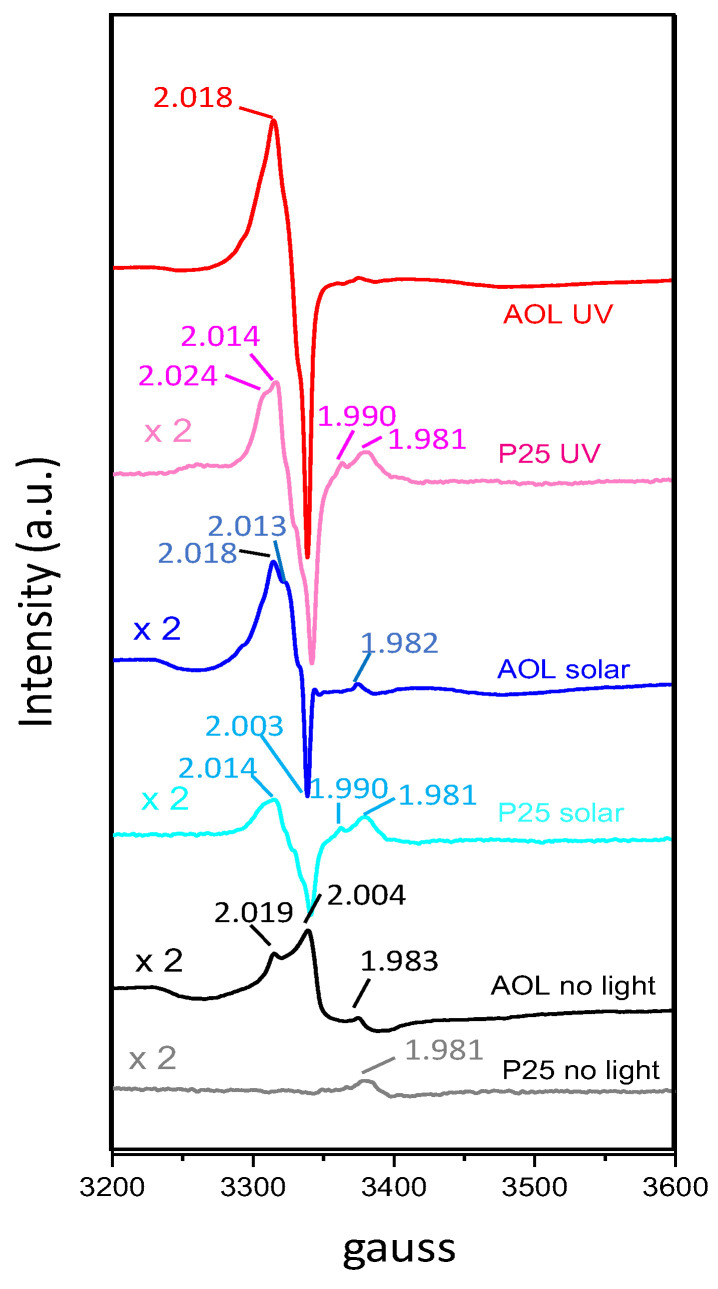
EPR spectra of Evonik P25 and AOL with (UV or solar) and without irradiation.

Finally, some complementary electron paramagnetic resonance (EPR) experiments were carried out. Results are summarized in Figure 7. EPR signals at g < 2 are typically associated to electron trapping sites, such as Ti^3+^, whereas those with g > 2 are ascribed to hole trapping sites such as oxygen radical anion species O^•—^ [46]. EPR spectrum of AOL without illumination presented signals at g = 1.983, 2.004 and 2.019 which can be attributed to Ti^3+^, single ionized oxygen vacancy F^+^ centers (also referred to as colored centers) and O^•—^ species (resulting from reaction of Ti^3+^ with adsorbed oxygen), respectively [49]. In the case of P25 electron trapping sites are slightly shifted to lower g values (g = 1.981) which is indicative of the presence of rutile [46]. N atoms can also contribute to the signal at g = 2.004 as nitroxide radicals NO• [50]. 

All in all, a comparison of EPR spectra without illumination of AOL and P25 suggests a higher presence of surface defects in AOL (more intense EPR signals), which is consistent with the larger amount of oxygen vacancies and Ti^+3^ species observed in XPS results and the redshift of light absorption observed for AOL.

Illumination of samples results in the excitation of electrons from the valence to the conduction band, thus leading to electron and hole trapping sites (Ti^3+^ and e.g., O^•—^ at g < 2 and g > 2, respectively). If signals obtained for catalysts under visible and UV irradiation in the region of g > 2 are compared, intensity and areas are higher for AOL than for P25. Moreover, the increase in signals from solar to UV irradiation is greater for AOL than for P25 (e.g., areas from 3250–3360 gauss multiplied for 3.5 and 2.8, respectively). This could suggest a lower electron-hole recombination in AOL, this difference being more relevant under UV irradiation.

The solids were tested for hydrogen production through photocatalytic reforming of glycerol both under UV and solar light in the devices shown in Figure 2A,B. The preliminary results are summarized in Table 2.

As can be seen, both under UV and solar irradiation, hydrogen production followed the sequence: AOL > P25 > anatase. In fact, under solar light, AOL produced 64% more hydrogen after 6 h than Evonik P25. Such an improvement was even higher under UV irradiation (143%). These results suggest that AOL better catalytic performance cannot be only associated to the observed redshift in light adsorption (i.e., a larger amount of useful irradiation to activate the process which would be important under solar irradiation) but to the more effective use of existing radiation, as a result of the reduced electron-hole recombination under both solar and UV radiation. These would be in line with the above-commented higher increase of EPR bands at g > 2 for AOL as compared to P25 when solar light is replaced by UV irradiation.

## 4. Conclusions

Olive leaves were used as templates to synthesize a titania-based solid (artificial olive leaf, AOL). AOL structure successfully replicated that of olive leaves (as evidenced by SEM) resulting in a 52 m^2^·g^−1^ material with macro, meso and microporous (nitrogen-isotherms). The solid is mainly constituted by titania in the anatase form (XRD and Raman) and showed a redshift in light adsorption as compared to reference anatase and P25 titania (as evidenced by UV-Vis). The solid also contains some other minor elements present in olive leaves such as nitrogen (1%) or carbon (1.6%). AOL was preliminarily tested as a catalyst for hydrogen production through glycerol photoreforming. The activity of AOL was 64% and 144% higher than that of Evonik P25 reference material under solar or UV irradiation. Both the redshift in light absorption and the reduction in electron-hole recombination (as suggested by EPR) could account for that. All in all, this manuscript widens the scope of potential applications of olive leaves, a major bioproduct in Andalusia, as a biotemplate for the synthesis of photocatalysts.

## Figures and Tables

**Table 1:** Elemental composition of AOL as determined by XRF. For comparative purposes, composition of olive leaves [27] has also been included.

Element	AOL (Weight %)	Olive Leaf (Weight %)
Ti	53.2	-
Ca	1.83	1.93
P	1.09	0.27
K	0.95	0.90
S	0.73	0.41
Si	0.38	0.12
Mg	0.12	0.21
Al	0.078	0.048
Fe	0.046	0.015
Zn	0.017	0.0023
Cl	0.0084	0.054
Sr	0.0077	0.0048

**Table 2:** Hydrogen photo-production on P25, AOL and anatase simples using UV or solar light.

Catalyst	Hydrogen Production (µmol/gcat)			
	UV (3 h)	UV (6 h)	Solar (3 h)	Solar (6 h)
P25	459.8	767.6	79.3	137.7
AOL	1035.2	1872.5	149.6	225.9
Anatase	372.8	669.9	52.4	73.3
